# Clinical Application and Educational Training for Pharmacogenomics

**DOI:** 10.3390/pharmacy8030163

**Published:** 2020-09-03

**Authors:** Jason W. Guy, Isha Patel, Julie H. Oestreich

**Affiliations:** 1 Department of Pharmacy Practice, College of Pharmacy, University of Findlay, Findlay, OH 45840, USA; 2 Department of Pharmacy Practice, Administration and Research, Marshall University School of Pharmacy, Huntington, WV 25755, USA; pateli@marshall.edu; 3 Department of Pharmaceutical Sciences, College of Pharmacy, University of Findlay, Findlay, OH 45840, USA; julie.oestreich@findlay.edu

**Keywords:** pharmacogenomics, pharmacy education, pharmacogenomics education, clinical outcomes

## Abstract

Pharmacogenomics—defined as the study of how genes affect a person’s response to drugs—is growing in importance for clinical care. Many medications have evidence and drug labeling related to pharmacogenomics and patient care. New evidence supports the use of pharmacogenomics in clinical settings, and genetic testing may optimize medication selection and dosing. Despite these advantages, the integration of pharmacogenomics into clinical decisions remains variable and challenging in certain practice settings. To ensure consistent application across settings, sufficient education amongst current and future healthcare providers is necessary to further integrate pharmacogenomics into routine clinical practice. This review highlights current evidence supporting clinical application of medications with pharmacogenomic labeling. The secondary objective is to review current strategies for educating health professionals and student trainees. One national organization predicts that most regions in the United States will soon contain at least one healthcare system capable of applying pharmacogenomic information. Applying genotype-guided dosing to several FDA-approved medications may help produce beneficial changes in patient outcomes. Identifying best practices for educating health care professionals and trainees remains vitally important for continuing growth of pharmacogenomic services. As pharmacogenomics continues to expand into more areas of healthcare, current and future practitioners must pursue and maintain competence in pharmacogenomics to ensure better outcomes for patients.

## 1. Introduction

Pharmacogenomics (PGx)—the study of how genes affect a person’s response to drugs—is a growing area in pharmacy and clinical practice [1]. Often, these changes occur with differences in metabolism or elimination of medications due to a particular drug–gene interaction. Drug–gene interactions describe how a patient’s genetic makeup can affect the outcomes of medication therapy in some way [2]. More complicated interactions can occur that involve drug–gene interactions with an additional drug or gene relevant to the medication [2]. For example, drug–drug–gene interactions can include a second medication that causes an effect on metabolism or elimination of the primary drug. Separately, an individual’s genotype can also impact the overall metabolism and/or elimination of the primary medication [2]. This added genetic effect can enhance or diminish the impact of the original drug–drug interaction. These interactions can be quite complex, and require health care professionals to remain up to date with the latest knowledge regarding pharmacogenomics.

According to the FDA, there are over 175 medications that currently contain drug labeling information related to pharmacogenomics [3]. This information can be clinically relevant to help predict responders, guide medication dosing, reduce side effects and tailor medications to promote effective therapeutic care [3]. In addition to classic pharmacogenomic markers associated with metabolizing enzymes and other germline variations, many of the recent entries cover companion tests for oncology-related medications. These genetic tests for somatic mutations are often required for drug selection and/or dosing of cancer treatments. These somatic examples are not usually incorporated into discussions of pharmacogenomics, and will not be further addressed in this review. Nonetheless, staying familiar with all medications that have actionable genetic information can be vital to enhance patient outcomes.

Actionable pharmacogenomics typically refers to germline PGx information that can be leveraged to increase efficacy, reduce safety risks, or change outcomes associated with medication therapy. Medications may or may not have pharmacogenomic information associated with them, and some medications with pharmacogenomic labeling may not contain information that is actionable—or that would change outcomes for a patient [4]. Many studies have evaluated the overall clinical impact of pharmacogenomic testing [5]. 

In addition, optimal integration of PGx will require health care professionals who are adequately prepared to manage patients’ drug regimens based on traditional clinical information and genetic test results. The interpretation and implementation of pharmacogenomics builds on routine drug therapy for patients and allows pharmacists to apply their deep understanding of pharmacotherapy and pharmacology. With these foundational skills, pharmacists possess the ability to provide the added service of pharmacogenomics to optimize patient therapeutic strategies. The ability to view pharmacogenomic data in context with a patient’s other medications is essential to provide clear and comprehensive recommendations that enhance patient care. Hence, the profession of pharmacy is uniquely positioned with opportunities to make a clinical impact in the area of pharmacogenomics.

Optimal integration of PGx in the curricula imparted to pharmacy students will adequately prepare them to manage patients’ drug regimens based on traditional clinical information and genetic test results. Training for pharmacogenomics begins at Colleges of Pharmacy where pharmacists are trained to handle medication related issues and coordinate care for patient medications. However, many curricula did not incorporate the additional consideration of pharmacogenomic services until recently. Therefore, healthcare professionals may require additional training either during a professional program or post-graduation to better prepare for managing drug regimens using pharmacogenomic data. The recent expansion of educational programs provided to pharmacists and other health care professionals helps enhance competency in PGx. 

This review will identify current evidence related to pharmacogenomics and the associated clinical outcomes from several studies across multiple settings. Additionally, the review will summarize some of the latest trends used to educate student pharmacists and licensed pharmacists in the area of pharmacogenomics.

## 2. Clinical Outcomes

The application of pharmacogenomics into real-world practice has been a slow, gradual process over the last couple of decades. Importantly, approximately 3% of all Americans carry a high-risk clinically actionable genotype for at least one medication when considering patients prescribed one of five medications or classes of medications—clopidogrel, warfarin, statins, thiopurines, or tacrolimus [6]. This represents a large number of individuals who could benefit from PGx interventions by pharmacists on a population level [6]. However, there is conflicting evidence in how pharmacogenomics should be implemented in clinical practice. Over time, alternative strategies such as replacing the agent or increasing monitoring have reduced the relevance of many actionable PGx examples with a high level of evidence. As the availability of PGx testing continues to spread and costs decrease, one considerable challenge relates to the possible reintegration of medications with actionable pharmacogenomic data. Furthermore, guidelines from therapeutic organizations do not universally endorse PGx testing, but sometimes provide recommendations for when pharmacogenomics may help treat a patient [5]. These guidelines can identify optimal candidates for pharmacogenomic testing that enhance patient outcomes. 

Several hospitals and academic medical centers routinely collect and apply PGx testing, and in some cases have done so for many years. Indeed, the American Society of Health-Systems Pharmacists predicts most regions in the United States soon will have a PGx hospital program [7]. However, the integration of PGx into unaffiliated ambulatory care settings and community pharmacies has remained more of a challenging issue, and thus far has advanced mostly in early adopter clinics and pharmacies. In the future, PGx opportunities may continue to arise in many settings, which could allow expanded access for patients. Ongoing research in this area will help determine the prospect of pharmacogenomics in unique settings. One such initiative—the ‘’1200 Patient Project’’—was designed to determine feasibility of outpatient pharmacogenomic services [8]. This project enrolled patients in an outpatient setting with preemptive pharmacogenomic testing, which allowed for storage of patient results prior to use as part of the therapeutic management plan and avoided waiting for results for each individual medication [8]. For 85% of patient visits in the study, relevant pharmacogenomic information was available for at least one drug in each patient’s medication list, thus showing the frequency and potential impact of pharmacogenomics for patient related outcomes [8]. 

Patient outcomes have been examined in a variety of clinical areas, and many studies have focused on genotype-guided dosing versus traditional dosing protocols. In particular, several psychiatric medications contain actionable PGx information relevant to patient outcomes. The recently completed GUIDED trial investigated patient outcomes for individuals with major depressive disorder (MDD) who had at least one prior medication failure [9]. Dosing for patients in the trial followed either genotype-guided medication selection and dosing or standard prescribing [9]. The results of the study showed that rates of response and remission improved in the genotype-guided therapy group; however, overall symptoms were not significantly improved [9]. Based on these results, a second trial was conducted for a subgroup of patients from the original study (~70% patients) who were taking medications with gene–drug interactions and who were most likely to benefit from clinical application of pharmacogenomics [9]. In this subgroup, patients in the genotype-guided dosing group showed significant improvement in major depression symptoms at 8 weeks, and those benefits continued to week 24 of the study [9]. The results of this study highlight the benefits of genotype-guided dosing for patients with depression who have failed a medication in the past. 

Additional studies in the area of psychiatry support the benefits of genotype-guided dosing for other common disease states. For example, Bradley et al. demonstrated that pharmacogenomic-based dosing improved response rates and remission rates compared to standard dosing protocols for anxiety [10]. The study also found that patients with PGx dosing decreased their Hamilton scores for anxiety more than the standard dosing group [10]. Another study involving a pharmacogenomic-guided dosing protocol in neuropsychiatric conditions demonstrated decreased adverse events when compared to patients treated with standard dosing protocols [11]. This study included a diverse set of disease states—anxiety, depression, ADHD, and psychosis—and medications such as benzodiazepines, SSRIs, antipsychotics, and stimulants. 

Genotype-guided dosing may also deliver value for select patient settings and outcomes. In the field of infectious diseases, pharmacogenomic-guided dosing helped reduce the number of patients with subtherapeutic concentrations of voriconazole at days 5–7 of antifungal prophylaxis therapy [12]. In a separate study, pediatric patients treated with voriconazole benefitted from genotype-guided dosing for antifungal prophylaxis in allogeneic hematopoietic stem cell transplantation (HSCT) [13]. Patients assigned to the genotype-guided dosing group reached the target drug concentration range in 6.5 days, compared to 29 days in the standard dosing protocol (*p* < 0.001) [13]. However, the above results focused on surrogate endpoints related to voriconazole kinetics rather than clinical endpoints. Despite this limitation, patients achieved therapeutic drug concentrations sooner, thus improving the chances of effective antifungal therapy in various patient groups. 

Research done in the field of cardiovascular disease has shown promising results as well. Recently, Syn et al. evaluated pharmacogenomic-based dosing vs. traditional dosing for warfarin in patients with Asian ethnicity [14]. Pharmacogenomic-guided dosing reduced the number of dose titrations needed in the first two weeks of warfarin therapy; however, the time within the therapeutic range did not change [14]. Similarly, a meta-analysis evaluating warfarin therapy found no difference in the time in therapeutic range between the pharmacogenomic-guided dosing and standard dosing protocols [15]. The above studies showed that pharmacogenomic-based dosing shortened the time to achieve a maintenance dose (supporting a role for PGx in surrogate measures) and also reduced the risk of adverse effects and major bleeding (Table 1) [15].

Other international studies investigating PGx outcomes also add to the literature and guidance on the potential beneficial role of PGx [16]. For example, a study in the Netherlands found that approximately 18% of eligible patients elected to receive genotyping [16]. Out of these patients, approximately 90% of them had at least one actionable PGx test result [16], indicating that many patients could benefit from widespread PGx testing [16]. 

While outcomes data play a vital role in the application of pharmacogenomics to patient care, cost considerations associated with pharmacogenomics cannot be ignored. High costs and inconsistent reimbursement procedures have hampered the routine use of pharmacogenomics in clinical practice. Over the last decade, some of these costs have decreased, and researchers have now started to evaluate the cost-effectiveness of pharmacogenomics in clinical settings. A systematic review by Zhu et al. determined that pharmacogenomic-guided treatment for clopidogrel and statins was cost-effective in the majority of situations [17]. However, other situations and medications had inconclusive evidence for the use of pharmacogenomics. For example, warfarin demonstrated conflicting evidence for genotype-guided dosing, as half of the studies reviewed showed mixed results or no cost effectiveness. Outside the cardiology field, a separate study found that, compared to standard thiopurine dosing, genotype-guided dosing reduced side effects and did not increase costs associated with care in inflammatory bowel disease patients with a thiopurine S-methyltransferase variant [18]. 

However, to date, the results remain mixed regarding cost effectiveness when utilizing pharmacogenomic data. Further implementation of PGx in routine clinical practice will require novel and sustained reimbursement options going forward. Additionally, there are few FDA-approved PGx platforms, which can provide a barrier to widespread testing availability. These reimbursement and regulatory issues pose a challenge to the implementation and utilization of PGx testing for clinical use.

Health professionals need knowledge of actionable PGx data and confidence to make therapeutic interventions with the data in order to enhance patient outcomes. To increase PGx abilities, pharmacists must receive adequate education and training in this area, through continuing education as a healthcare professional and formal training as a student pharmacist through college curricula. 

## 3. Educational Outcomes

One barrier to the implementation of pharmacogenomics into routine clinical practice has been the education of pharmacists and other health care professionals. In addition, health care providers perceive a lack of evidenced-based recommendations that are sufficiently useful for their ordinary workflow, and therefore may be less confident and unlikely to make recommendations regarding therapy. As a result, academicians and professional pharmaceutical organizations have developed several options to educate both pharmacists and student pharmacists on the basic tenets and application of pharmacogenomics. These ongoing and emerging programs help facilitate the implementation process and utility of pharmacogenomics in the field [19,20,21,22,23,24].

Importantly, the American Society of Health-Systems Pharmacists (ASHP) has charged pharmacists with spearheading the clinical application of pharmacogenomics [7]. ASHP specifically called on Boards of Pharmacy and Colleges of Pharmacy to include curricula on PGx to enable pharmacists to recommend appropriate genetic testing, tailor drug therapy, and provide education to other healthcare professionals [7]. In the future, all pharmacists will need some baseline level of PGx training, with some requiring advanced training in the area [7]. Thus, best practices endorse training for both pharmacists and students to advance pharmacist skills in the future. 

### 3.1. Pharmacist-Based Training

Access to foundational and advanced knowledge about PGx and making training opportunities available will ensure pharmacists maintain knowledge as new technology and processes emerge to further enhance clinical practice. Leveraging flexible methodologies and learning strategies, including asynchronous formats, will allow pharmacists to quickly and easily access information. 

One way to enhance training in the area of PGx is through easily accessing continuing education courses. Formea et al. developed a continuing education course to help enhance pharmacist knowledge about PGx, and saw an overall knowledge increase by 7% among pharmacists [25]. Unfortunately, even with this slight improvement, pharmacists still only received an average score of 53%. This low average score raises concerns for the profession deemed critical to the advancement of PGx, and indicates the great need for repeated exposure and increased quantity of resources available. Another study found that a separate continuing education program improved pharmacist knowledge and attitudes regarding PGx, including a greater willingness to implement PGx into their practice sites [26].

Recognizing the importance of uptake outside of pharmacy, the Mayo Clinic described a process to improve education among all health care providers [27]. This study stressed the value of interprofessional education and collaboration to solve problems in the organization regarding PGx [27]. Pharmacists in the institution were provided online training regarding the latest developments in PGx [27]. These trainings resulted in significant improvements by pharmacists (45% increase in post-test correct scores) after completing the online modules [27]. After training, the pharmacists worked with actionable PGx data and offered support to prescribers to enhance patient care [27]. If the pharmacist was unable to effectively provide advice regarding the data, the case was escalated to a senior member of the pharmacogenomics team for further clarification [27]. Overall, this model shows the potential usefulness of PGx when integrated across multiple disciplines in the healthcare system.

Advanced educational opportunities beyond continuing education also are needed to offer pharmacists specialized training in PGx. In recent history, several organizations and universities have created post-graduate opportunities to help pharmacists increase their knowledge and skills regarding PGx. In addition, several certificate programs now exist, including continuing education courses and more traditional academic credit-based programs (Table 2) [19,20,21,22,23,24]. Some institutions even offer master’s degrees in pharmacogenomics that can help pharmacists integrate pharmacogenomics [22,28].

### 3.2. Student-Based Training

The field also needs Colleges of Pharmacy to help improve the education of future pharmacy professionals so they can adeptly provide evidence-based therapeutic recommendations with pharmacogenomic data. The latest guidelines from the Center for the Advancement of Pharmacy Education (CAPE) and the Accreditation Council for Pharmacy Education (ACPE) emphasize the need to train student pharmacists in regards to PGx as part of evidence-based decision making [29,30]. The effort to fill knowledge gaps of student pharmacists is evident from the number of schools that have developed content focused on PGx in their curriculum. Notably, a 2019 survey of schools of medicine, pharmacy, nursing, and health professions found that 87% of programs offered at least some training in PGx [31]. Most of the content from the surveyed schools was delivered as part of pharmacology curricula or as an elective course in the program [31]. Approximately 50% of the schools who did not offer PGx content planned to offer it within the next 2 to 3 years [31].

While many schools include PGx content in their curricula, the amount of content and the nature of delivery varies among programs. Specifically, a 2019 analysis of PharmD curricula in the United States observed that 42% of pharmacy schools offered a required core course in PGx [32]. Another 8% of pharmacy schools established at least one elective course in the area of PGx [32].

Due to advancements in the area of PGx and the potential for tailored therapeutic regimens, some Colleges of Pharmacy have further emphasized the importance of these new courses to improve student knowledge in the area. A study by Marcinak et al. found that students who participated in a PGx elective course were more comfortable and confident in the application of PGx clinically [33]. Most students in this study also thought that the PGx elective course added value to their overall education [33]. PGx-based elective course offerings can help prepare students for practice-based settings by improving their confidence when faced with complex clinical situations. 

Data suggests that course electives integrate PGx content into curricula and improve student performance when instructors apply active learning strategies. For example, activities performed in a lab experience improved student learning by actively engaging students with PGx in an experiential exercise [34]. Additionally, PGx courses that include unique mechanisms to engage students also help to improve their confidence. Galvez-Peralta et al. designed a PGx course that included role playing situations where students assumed the role of a pharmacist to simulate patient communication [35]. In this same course, students also participated in a class debate regarding the ethics of PGx and personalized medicine [35]. These learning activities increased students’ confidence in discussing PGx with other health care providers [35]. 

A form of active learning that has gained traction in many curricula over the last decade is experiential learning. This type of learning often involves students or trainees developing skills with a hands-on approach, generally through an integrated experience, practice, or event. A recent study applied personal genomic testing into a course in the pharmacy curriculum [36]. During this course, students who conducted their own voluntary PGx test displayed patient empathy, enhanced knowledge of genomic testing, and increased confidence in interpreting test results [36]. Other similar studies that either incorporated personalized genotyping results into an online elective course or as part of a first-year pharmacy program demonstrated increased student confidence and knowledge associated with PGx [29]. The success of these collective projects helps highlight potential educational benefits for future pharmacists [37]. Another group of researchers tested the integration of software into a PGx elective course that allowed students to obtain experiential training with simulated prescriptions and gene–drug interactions [38]. This hands-on simulation was valued by students whose feedback was captured through focus groups, suggesting that it could serve as a useful tool for helping students understand pharmacogenomic data [38].

Several universities have created post-graduate opportunities in PGx to help professionals seek advanced pharmacogenomic training [28,39,40]. Some programs prepare residents for faculty positions in PGx or clinical positions that focus on PGx in health care settings, government entities, or pharmaceutical industry [28]. Other programs allow pharmacy graduates to complete a master’s degree and receive additional board credentials or provide PGx fellowships [39,40].

Beyond formal education, pharmacists will need skills to access PGx information in real time when handling clinical situations. To meet that need, several clinical resources offer evidenced-based guidelines for both education and clinical practice. For example, the Clinical Pharmacogenetics Implementation Consortium (CPIC) guidelines provide recommendations regarding actionable PGx data [5]. CPIC resources also include a listing of genes with potential drug interactions as well as an evidence rating to help ascertain the documentation and relevance of each interaction [5]. Another educational resource is the PharmGKB database, which contains lists of medications with PGx labeling information and serves as a curator of gene–drug connections and the literature [41]. In addition, the Food and Drug Administration (FDA) has created a list of medications with pharmacogenomic biomarkers in drug labeling. This list serves as a useful resource to identify medications with potential implications in the area of PGx; however, some of the listed medications did not have evidence suggesting an impact on clinical outcomes [3]. Hence, recently the FDA provided an additional resource that contains information related to the level of evidence associated with PGx for each medication. This list separates medications into different categories, including (1) data supporting therapeutic management recommendations, (2) data indicating potential impact on safety or response, and (3) data indicating a potential impact on pharmacokinetics only [42].

While these new opportunities, resources, and pedagogy have helped integrate PGx more fully, further work is needed to promote PGx in the future. A large number of pharmacists still believe they cannot accurately apply results of PGx tests to recommend drug therapy selection, dosing, or monitoring [43]. Therefore, additional studies should consider innovative forms of PGx education to improve overall knowledge of future pharmacy professionals, current pharmacy professionals, and other healthcare professionals. A summary of current literature involving training methodologies used for PGx training is shown in Table 3.

## 4. Conclusions

Pharmacogenomics is a growing field in healthcare that tailors an individual’s medication therapy with genetic testing to improve patient care. Several notable studies have demonstrated the potential benefits of genotype-guided dosing protocols to enhance patient efficacy and safety. However, while some medications are well-suited for full integration of actionable PGx data into the clinical decision process, other medications may not show much benefit. As more information becomes available regarding drug–gene interactions, pharmacists should understand the current evidence in the literature, and its impact on clinical practice and the education of fellow healthcare providers. Numerous opportunities are available for future and current pharmacy professionals to obtain their PGx training through continuing education and advanced training experiences. Adding the elements of active and experiential learning in PGx courses in Colleges of Pharmacy can aid in developing broader training exercises to enhance trainee confidence and learning about PGx.

## Figures and Tables

**Table 1:** Clinical Outcomes Associated with Pharmacogenomic Dosing Studies.

Authors of Study	Medications Involved	Design	Summary of Result
Thase et al. [9]	Medications to treat depression (specific medications not listed)	Subgroup of patients with MDD and gene–drug interaction	Genotype-guided dosing significantly improved symptoms at week 8, compared to standard dosing
Syn et al. [14]	Warfarin *	Pharmacogenomic-based dosing vs. traditional dosing for warfarin in patients with Asian ethnicity	Decreased dose titrations with pharmacogenomic-based dosing, but no significant difference in time within the therapeutic range
Shi et al. [15]	Warfarin *	Warfarin therapy with pharmacogenomic-based dosing or standard dosing	Time in therapeutic range did not differ. PGx dosing shortened time to a stable maintenance dose and also reduced the risk of adverse effects
Bradley et al. [10]	Medications to treat anxiety (specific medications not listed)	Pharmacogenomic-based dosing vs. traditional dosing for patients with anxiety	Improved score for anxiety with pharmacogenomics
Patel et al. [12]	Voriconazole ^+^	Pharmacogenomic-based dosing vs. traditional dosing for voriconazole	Fewer patients with subtherapeutic concentrations in the genotype-guided dosing group
Teusink et al. [13]	Voriconazole ^+^	Pharmacogenomic-based dosing vs. traditional dosing for voriconazole in patients with hematopoietic stem cell transplantation (HSCT)	Patients reached target therapeutic concentrations sooner in the genotype-guided dosing group
Sluiter et al. [18]	Thiopurine	Genotype-guided vs. standard thiopurine dosing in patients with inflammatory bowel disease	Genotype-guided dosing reduced risk of side effects and did not increase costs

* Contained on FDA list for pharmacogenetic associations for which the data support therapeutic management recommendations; ^+^ contained on FDA list for pharmacogenetic associations for which the data demonstrate a potential impact on pharmacokinetic properties only.

**Table 2:** Training Programs for Pharmacogenomics.

Organization	Program
Mayo Clinic [24]	Pharmacogenomics Certificate Course
American Society of Health Systems Pharmacists [21]	Pharmacogenomics Certificate
National Association of Chain Drug Stores [23]	Community-Based Pharmacogenomics Certificate Program
University of Colorado [19]	Graduate Certificate Program
Shenandoah University [20]	Pharmacogenomics and Personalized Medicine Certificate
Manchester University [22]	Graduate Certificate or Master’s Program
University of Florida [28]	Graduate Certificate or Master’s Program

**Table 3:** Educational Methodologies for Pharmacist and Student Pharmacist Training.

Authors of Study	Method	Summary of Result
Formea et al. [25]	Continuing education course	Increased knowledge scores
Kuo et al. [26]	Continuing education course	Improved knowledge and attitude scores
Formea et al. [27]	Online training modules	Improved post test scores
Weitzel et al. [29]	Personalized genomic testing	Improved confidence and knowledge in pharmacogenomics
Galvez-Peralta et al. [35]	Role playing situation and class debate	Improved confidence in ability to discuss pharmacogenomics with other health care providers
Adams et al. [36]	Personalized genomic testing	Increased confidence in understanding test results
Springer et al. [38]	GeneScription software	Simulated processing prescriptions with gene–drug interactions
